# Supplementary figures and images for: WormQTL2: an interactive platform for systems genetics in Caenorhabditis elegans
Source: Database (Oxford). 2020 Jan 21;2020:baz149. doi: 10.1093/database/baz149 (PMC6971878; doi:10.1093/database/baz149)

## Slide 1
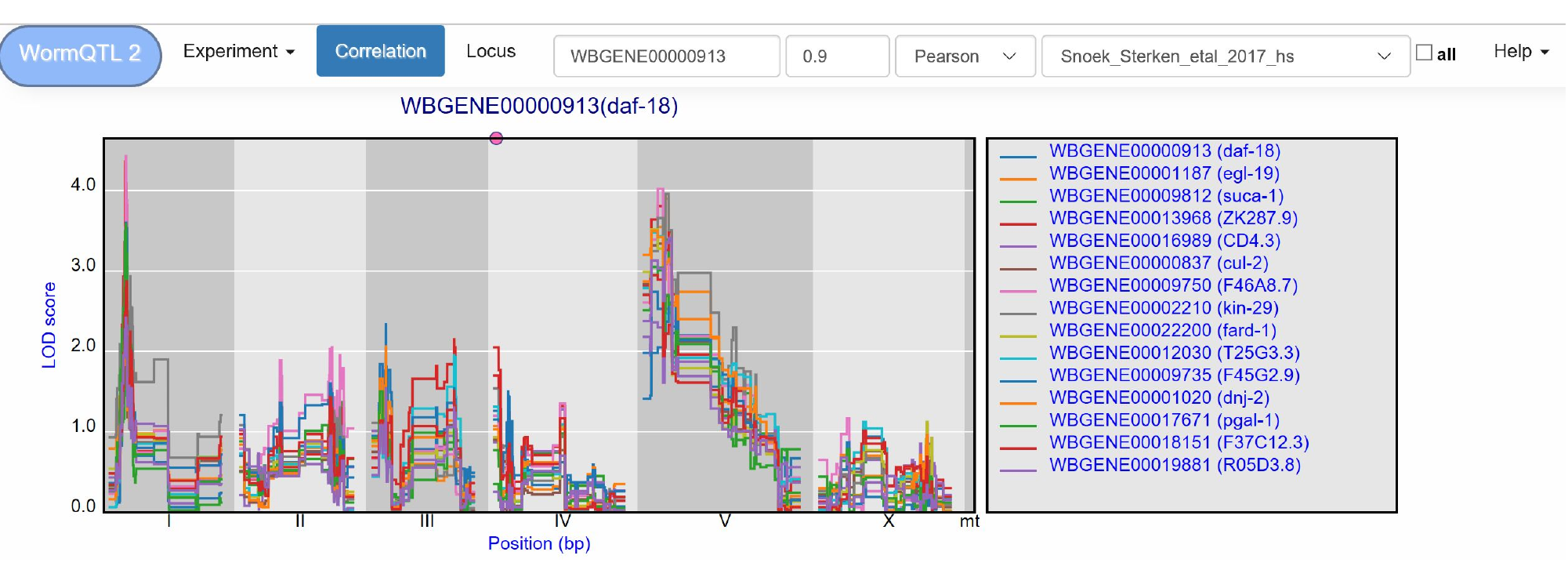

Supplement: Supplement_Figure_1_baz149 [file supplement_figure_1_baz149.pptx]

## Slide 1
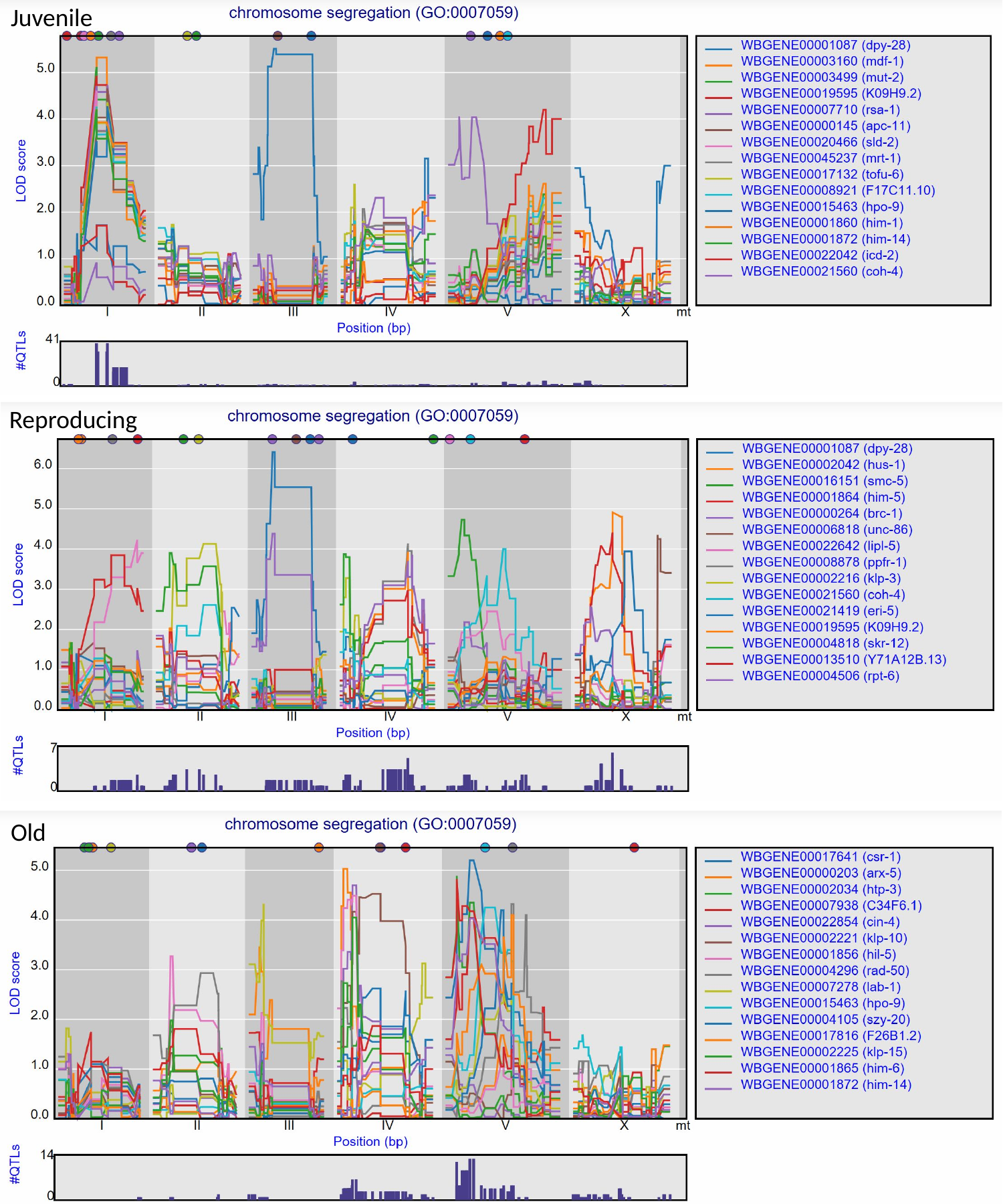

Juvenile
Reproducing
Old

Supplement: Supplement_Figure_2_baz149 [file supplement_figure_2_baz149.pptx]
